# Role of Lignin in Hot-Pressing of Paper: Insights from Molecular Simulations and Experiments

**DOI:** 10.1021/acs.biomac.5c00872

**Published:** 2025-08-01

**Authors:** Patric Elf, Amanda Mattsson, Antti Paajanen, Jukka A. Ketoja, Gunilla Pettersson, Jose Luis Sanchez-Salvador, Angeles Blanco, Carlos Negro, Per Engstrand, Mikael S. Hedenqvist, Fritjof Nilsson

**Affiliations:** † School of Engineering Sciences in Chemistry, Biotechnology and Health, Fibre and Polymer Technology, 7655 KTH Royal Institute of Technology, SE-100 44 Stockholm, Sweden; ‡ FSCN Research Centre, Mid Sweden University, 85170 Sundsvall, Sweden; § VTT Technical Research Centre of Finland Ltd., Box 1000, FI-02044 VTT Espoo, Finland; ∥ FibRe Centre for Lignocellulose-Based Thermoplastics, 7655 KTH Royal Institute of Technology, SE-100 44 Stockholm, Sweden; ⊥ Department of Chemical Engineering and Materials, Faculty of Chemistry, 16734 Universidad Complutense de Madrid, Avda. Complutense s/n, ES-280 40 Madrid, Spain

## Abstract

Improving the mechanical properties of wood and paper is crucial for enhancing their performance in structural and packaging applications. A particularly effective method for increasing strength is hot-pressing, where lignin softening has been proposed as a key mechanism underlying improved fiber bonding. In this study, we investigated the deformation behavior of Norway spruce lignin across temperatures of approximately 25–300 °C and moisture contents of 0–25 wt % using molecular dynamics simulations and paper hot-pressing experiments. We simulated key mechanical paper properties, including Young’s modulus, glass transition temperature, and the diffusivity of water and lignin chains. Experimental results showed a pronounced increase in wet strength above 175 °C, which correlated with lignin softening and enhanced fiber–fiber bonding in the simulations. Our findings highlight the ability of molecular simulations to elucidate the mechanisms of lignin-driven bonding and provide a foundation for optimizing the use of lignin-rich materials in various applications.

## Graphical abstract

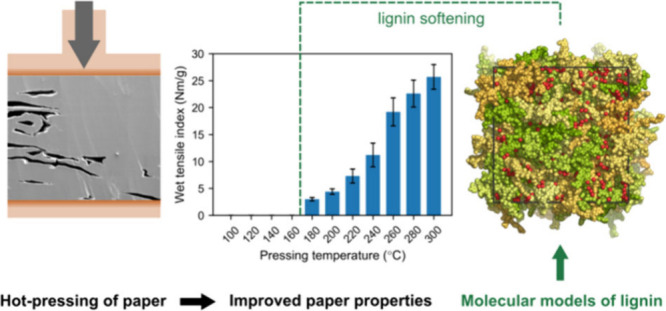

## 1 Introduction

The transition to more sustainable materials would greatly benefit from using the full potential of natural lignocellulosic fibers. Traditional fiber products, such as paperboard, rely on the inherent properties of cellulose fibers, including their high stiffness and strength, hygroscopicity, wet-moldability, and effective bonding after drying. However, many emerging fiber applications require the fiber network to exhibit significant wet strength, which cellulose alone cannot provide. This limitation has led several research groups^1–11^ including Joelsson et al.^12^ to investigate nonconventional production conditions in which other fiber components, such as lignin and hemicellulose, can be activated to provide the missing functionality. Recent experimental studies^12–16^ indicate that hot pressing of paper webs drastically enhances the wet strength of fiber networks when the fibers contain a sufficient amount of lignin. Moreover, this effect becomes more pronounced with increasing pressing temperature and duration. Despite these advancements, a fundamental understanding of the underlying molecular mechanisms remains lacking, hindering the optimization of hot-pressing operations. Mattsson et al.^18^ proposed that lignin interdiffusion at elevated temperatures could play a dominant role in the formation of wet-strong interfiber bonds. However, to fine-tune the process conditions for a given application, these mechanisms should be understood at a quantitative level, which means linking molecular-scale phenomena with experimental observations with varying parameters such as temperature and moisture content.

In this study, we take a first step toward describing fundamental aspects of lignin softening^8,9,19–21^ and mobility by introducing a molecular model for softwood lignin. This model is then used to simulate lignin molecular dynamics in the condensed phase across a range of temperatures and moisture contents. The simulations allow us to predict key material properties indicative of thermal softening behavior, including elastic and bulk moduli, the glass transition temperature, and chain diffusivity. We also obtain predictions for the structure and mobility of water within the amorphous polymer. A key consideration in the model development is the variability in lignin composition due to the botanical origin and extraction method. Our lignin model is based on the spectroscopic and chromatographic analysis of Balakshin et al.,^22^ which describes the chemical structure of spruce milled-wood lignin in terms of the relative abundance of different internal and terminal substructures, interunit linkages and functional groups, and the molecular weight distribution and degree of branching. While alternative lignin structures have been proposed in the literature^23^ and used in molecular dynamics studies,^24–26^ our model is particularly well-suited for mimicking native spruce lignin. Although there is still no consensus on the structure of native softwood lignin, our branched molecular structure, which is based on experimental data, is a step forward from simplified small-molecule and linear chain models.

The aim of this study was to systematically correlate the experimental strength of hot-pressed paper sheets with molecular dynamics (MD) simulations of spruce lignin. Our simulation results were therefore compared with the experimental wet strength development observed for hot-pressed chemi-thermomechanical (CTMP) spruce paper webs. The comparison between predicted and measured properties suggests that dominant strength mechanisms originate from the molecular level and can thus be described through simulations on this scale. Providing a comprehensive quantitative description of all mechanisms involved within the fiber wall^27,28^ or during the actual hot-pressing operation would require a multiscale approach, which is beyond the scope of this work. However, since lignin usually softens at lower temperatures than crystalline cellulose decomposes (∼140–220 vs ∼320 °C),^29,30^ it was, for our purpose, sufficient to examine the lignin.

## 2 Experimental Methods

### 2.1 Hot-Pressing of Paper Sheets

An extensive experimental test series was conducted to investigate how the wet and dry strength of paper are influenced by different hot-pressing conditions, such as temperature, moisture content, and pressing time. Chemi-thermomechanical pulp (CTMP) of softwood spruce from the SCA Östrand mill (Timrå, Sweden) containing 46.9% cellulose, 25.7% hemicellulose, and 26.6% lignin (Klason) and with a CSF freeness of 420 mL was used for all experimental work. The fiber characteristics, including an average fiber width of 31 μm, an average fiber length of 1.46 mm, and a fines content of 34.5%, were determined using a fiber tester (L&W Fiber Tester Plus ABB Group, Sweden) in accordance with the ISO-16065-2:2014 standard. The CTMP pulp was first prepared according to ISO 5263-3, followed by producing hand-sheets of different moisture content levels with a Rapid Köthen sheet former (PTI, Austria) according to ISO 5269-2. The sheets had a grammage of 100 g/m^2^ with moisture content (mc) between 7 and 25 wt %. For the moist samples, with moisture content above 7%, the levels were adjusted by stopping the drying process in the sheet former before the sheets were fully dry. The dry sheets, with a moisture content of 7 wt %, were stored at (noncontrolled) room climate.

The hot-pressing was done with a planar press installed in a hydraulic MTS machine (Figure SI1) with a capacity up to 100 kN and a pressing temperature of up to 300 °C. The advantage of this laboratory setup was the possibility of carefully controlling all process conditions except the room humidity. Two separate parts of the hot-pressing experiments were conducted. In the first part, the effects of pressing temperature (100 to 300 °C) and initial moisture content (7 and 25 wt %) were investigated using a pressing time of 3 s at 3.5 MPa, followed by a 6 s hold period at 0.1 MPa. In the second part, the effect of initial moisture content (7 to 25 wt %) was studied at a pressing temperature of 260 °C and pressing pressure of 3.5 MPa with pressing times of 1 and 5 s.

### 2.2 Microscopy

A high-resolution SEM (Tescan Maya3-2016, TESCAN, Czechia) was used for imaging the paper sheets prepared under different pressing conditions. The applied electron beam voltage was 3.00 kV, and the working distance to the sample, which ranged from 6 mm to 8 mm, was adjusted for each image to achieve an optimal image quality. The cross sections were prepared with an argon ion miller (Hitachi IM4000Plus, Hitachi High-Tech Co., Tokyo, Japan). All samples were prepared by sputtering them with a 5 nm iridium layer prior to imaging.

### 2.3 Physical Characterization

The pressed and unpressed sheets were characterized after conditioning them at 23 °C and 50% relative humidity for at least 24 h (ISO 187). Density was then determined from the measured weight and thickness and area (ISO 534 and 536). Finally, tensile strengths in dry and wet states (i.e., dry and wet tensile index) were measured according to ISO 1924 and 3781, respectively. Here, tensile index (Nm/g) refers to tensile strength (N/m) divided by the grammage (g/m^2^). Effectively, the same units are also obtained by dividing stress (N/m^2^) by density (kg/m^3^), which is usually called the specific strength.

## 3 Molecular Simulation Methods

### 3.1 Softwood Lignin Model

The starting point for modeling lignin behavior in softwood fiber walls at atomistic scale was to obtain a reasonable approximation for its chemical structure. For this purpose, we used the chemical analysis of spruce milled-wood lignin reported by Balakshin et al.^22^ Their work gives a statistical description of the lignin chemical structure based on combined spectroscopic and chromatographic evidence. Using information on molecular weight distribution and degree of branching and the relative abundance of different lignin moieties, it is possible to build condensed phase models that properly capture the diversity of the macromolecular chains. However, considering a broad molecular weight distribution would mean significant variation between individual nanometer-scale models. Therefore, we chose to represent softwood lignin as a collection of chains with a uniform molecular weight. The individual chains were given the chemical structure shown in Figure 1, which is identical with the representative structural model proposed by Balakshin el al.^22^ The chain consists of 26 repeat units, which corresponds to the observed number-average degree of polymerization.

**Figure 1:**
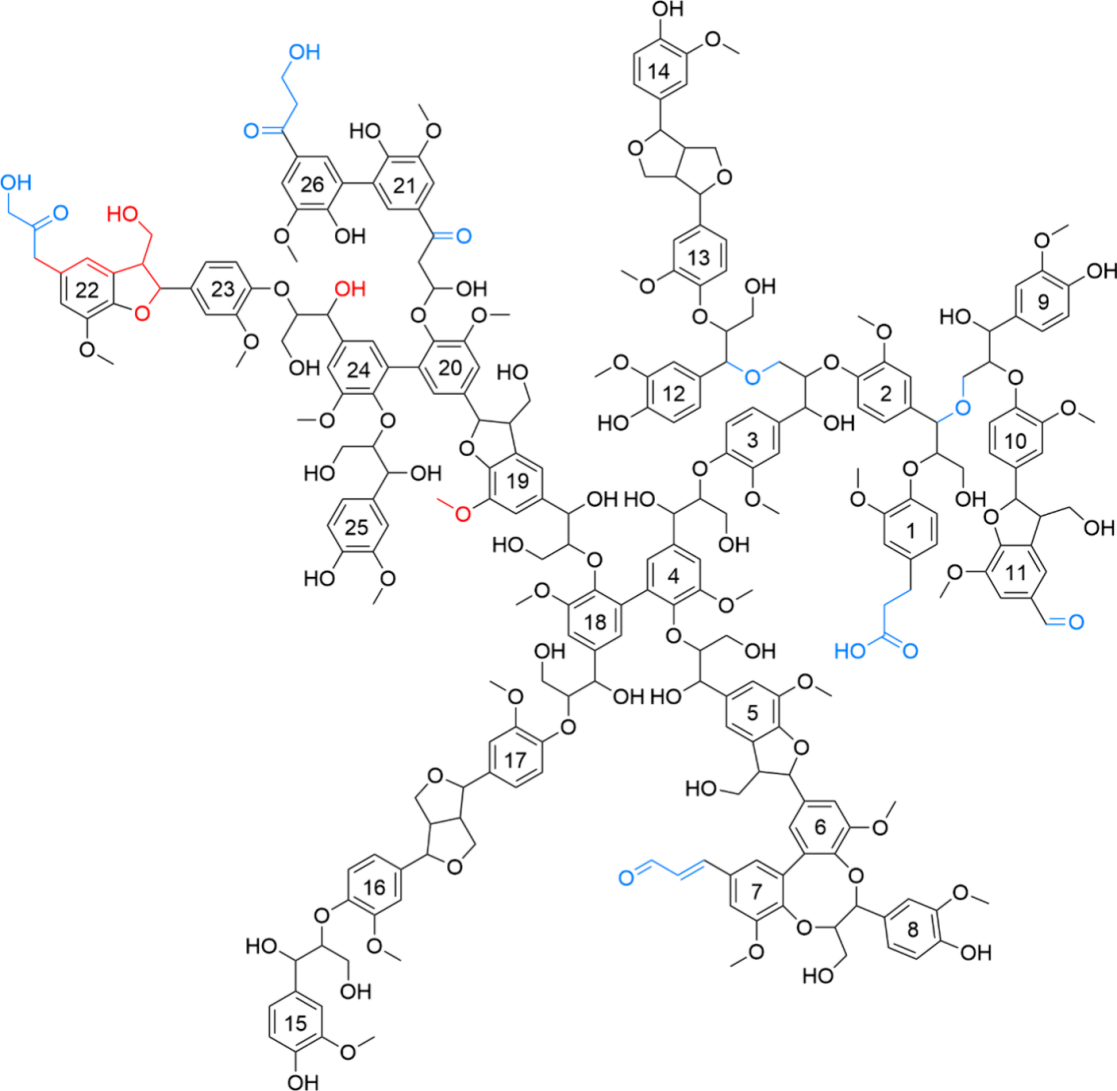
Skeletal formula of the used softwood lignin model, adopted from Balakshin et al.^22^ Blue color indicates linkages/terminal structures added to the LigninBuilder software, whereas red indicates substructures that were approximated with similar ones. The numbers inside the rings are repeat unit indices, as shown in Table 1.

Turning the chemical structure into a functioning molecular model requires a molecular force field that describes the interatomic forces and algorithms that create molecular geometries and topologies of the individual macromolecules. In this work, we used the CHARMM force field^31^ to describe the interatomic forces and a modified version of the LigninBuilder software^32^ to create the molecular geometries and topologies. The force field was extended with additional parameters, obtained using CHARMM-GUI^33^ to ensure compatibility with the implemented modifications.

LigninBuilder is a software tool for creating complex molecular models of lignin macromolecules. It is implemented as a VMD^34^ plugin and utilizes NAMD^35^ for energy minimization and structure optimization, which ensures that the generated lignin molecules adopt physically plausible conformations. The original program contains a large library of molecular geometry and topology templates for different monolignol units, interunit linkages, terminal units, and functional groups. These templates describe how the monolignol units bond to each other, and they also contain the necessary force field parameters for their intra- and intermolecular interactions. The tool facilitates molecular modeling of lignins by automating the generation of a wide array of lignin types. The LigninBuilder library, however, is missing some of the chemical substructures and linkages present in the chosen softwood lignin structure, particularly the aliphatic ether linkages that are involved in the branching points. Thus, several new linkages and terminal residues were added to LigninBuilder as shown in Table 1 and Figure 1. The modified tool was then used to create a molecular model that closely mimics the chosen softwood lignin structure. Some minor differences are still present, as indicated in Figure 1 by the red color. However, these differences are expected to have minimal impact on the mechanical characteristics of the lignin systems.

**Table 1:** List of Modifications That Were Added to LigninBuilder and Where These Modifications Were Implemented, as Seen in Figure 1

Modification	Repeat unit
α-O-γ-linkage	2–9, 12–3
γ-carboxyl	1
γ-aldehyde	7
α-aldehyde, removed β- and γ-carbons	11
α-ketone	21
β-ketone	22
α-ketone	26

### 3.2 Molecular Systems and Equilibration

All molecular systems were constructed using 128 molecules with the 26-monomer structure, with moisture contents ranging from 0 to 25 wt % in 5 wt % increments (Figure 2). The lignin molecules were randomly distributed within a 21 × 21 × 21 nm^3^ simulation box, after which water molecules were added at random locations. The TIP3P water model was used. These systems were then equilibrated by using a well-tested 21-step slow-decompression scheme, which first iterates between high and low temperatures with gradually increasing pressure, thereby compressing the simulation box. Thereafter, the pressure is gradually decreased so that the systems form equilibrated, densely packed systems at 300 K. Finally, the system is allowed to relax for 10 ns at 300 K temperature and 1 atm pressure, after which the density and potential energy terms are evaluated to ensure proper equilibration without noticeable drift.^36–38^ After the initial equilibration at 300 K, the systems underwent an additional 10 ns isothermal–isobaric (NPT) simulation at the other target temperatures (425, 450, 500, and 550 K). The resulting trajectories were subsequently analyzed to confirm the absence of any significant drift in terms of energy or density. The size of the equilibrated system was approximately 10 × 10 × 10 nm^3^. Raman spectroscopy has revealed a lignin-rich layer with a thickness of 0.1–0.5 μm between individual cellulose fibers,^28^ so the size of the simulation box is probably realistic.

**Figure 2:**
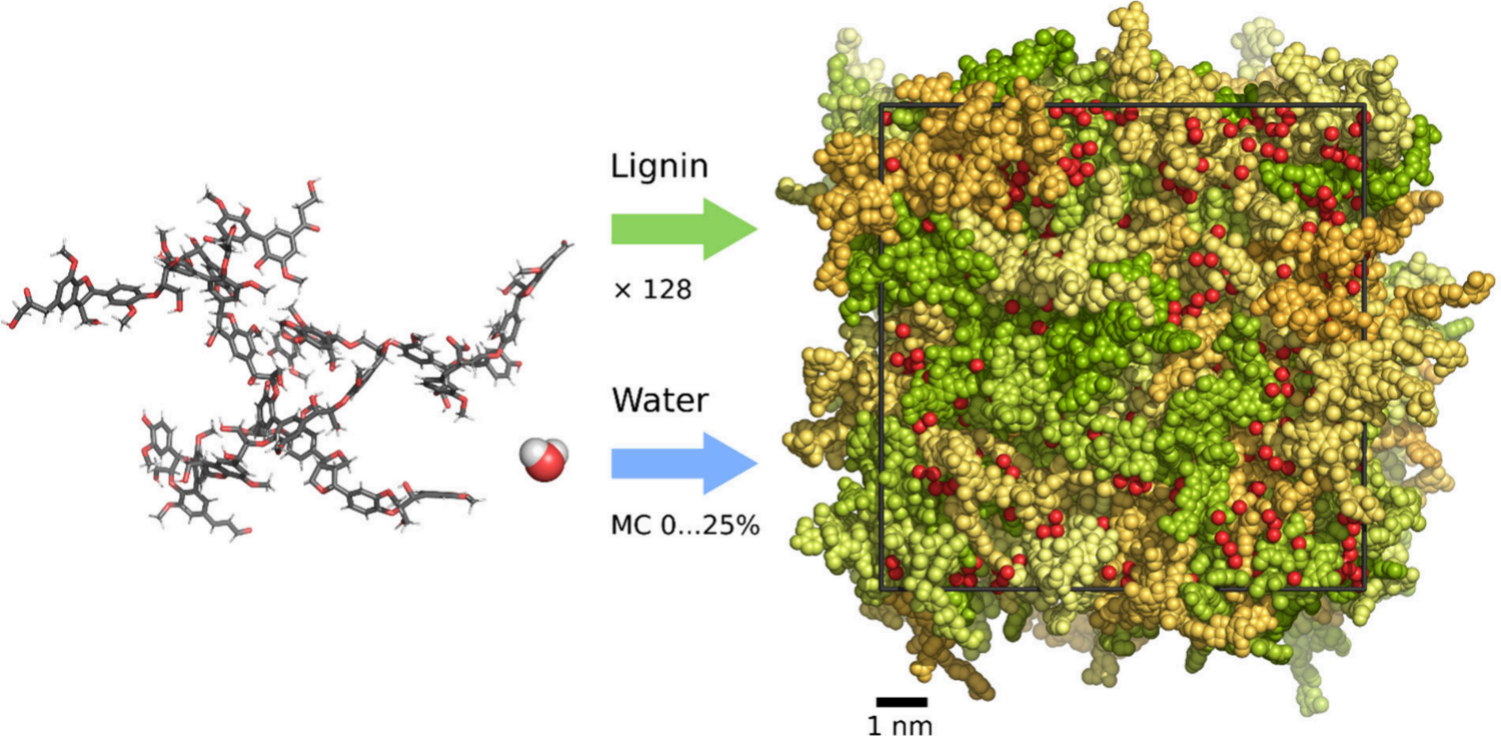
Schematic of how the condensed phase lignin models were built. 128 lignin molecules and 0–25 wt % water were inserted into a periodic cubic simulation domain, which was then equilibrated using an efficient 21-step annealing routine. Left: an individual lignin chain is shown using the stick representation of covalent bonds. Right: a condensed phase lignin model is shown with the lignin chains (green, yellow, orange) and the water oxygens (red) drawn using van der Waals spheres. The cubic simulation domain is outlined in black.

### 3.3 Glass Transition Temperature and Diffusivity

After the systems were equilibrated, they were subjected to stepwise cooling from 550 to 150 K. A 150 ns simulation was first carried out at 550 K, after which trajectory snapshots were taken at 110, 120, 130, 140, and 150 ns to obtain five parallel systems with distinct chain conformations. After this, the temperature of each system was decreased in 25 K steps at 10 ns intervals until 150 K was reached, producing an effective cooling rate of 2.5 K/ns. The simulations were carried out with 1 atm external pressure using a Parrinello–Rahman barostat and a velocity-rescale thermostat.

The glass transition temperature (*T*_g_) at each moisture content was primarily estimated by bilinear fitting against the temperature-density data averaged over five parallel trajectories. More specifically, the transition point was approximated by the crossover point of lines fitted to the low (150–225 K) and high temperature (500–550 K) linear regions of the temperature-density curves. The Williams–Landel–Ferry (WLF) equation (eq 1) was used to obtain the shift factor *a*_T_, which was used to make a rough extrapolation to estimate *T*_g_ at the laboratory-scale cooling rate of 10 K/min.^39,40^

$$\log\left(a_{T}\right)=\frac{C_{1}\left(T-T_{g}\right)}{C_{2}+\left(T-T_{g}\right)}$$ 1

The parameters *C*_1_ and *C*_2_ were obtained from an additional series of constant-rate cooling simulations, which are described in the Supporting Information.

Since the bilinear *T*_g_ method is sensitive to curvature in the density–temperature data, two additional methods were applied to assess *T*_g_. In the hyperbolic-*T*_g_ method,^41^ the density data is first fitted, as a function of temperature *T*, using a hyperbolic function to describe the density ρ­(*T*) (eq 2 and 3), where ρ_0_, *a*, *b* and *T*_0_ are adjustable fitting parameters. The second derivative of ρ is then calculated, and *T*_g_ is determined as the temperature corresponding to the minimum of this curve.

$$\rho \left(T\right)=\rho_{0}-a\left(T-T_{0}\right)-bH_{0}\left(T,T_{0},c\right)$$ 2

$$H_{0}\left(T,T_{0},c\right)=\frac{1}{2}\left(T-T_{0}\right)-\sqrt{\frac{{\left(T-T_{0}\right)}^{2}}{4}+e^{c}}$$ 3

In the *T*_g_-onset method, the onset of *T*_g_ is defined as the lowest temperature at which the deviation between the hyperbolic fit and the lower linear region of the bilinear fit exceeds a user-defined threshold, e.g., 2%. The *T*_g_-onset value is sensitive to the chosen threshold but unaffected by the curvature of the density–temperature curve at high temperatures. With a 2% threshold, *T*_g_-onset will be higher than the bilinear *T*_g_ when the *T*_g_-transition range is narrow and lower when it is broad. Since *T*_g_-onset indicates when a transition in thermal expansivity is initiated rather than when it is completed, it can give significantly lower *T*_g_ estimates than the other methods.

To obtain diffusivity estimates for the lignin chains and water molecules, the simulations were continued for 200 ns for two of the five parallel systems at temperatures of 300, 400, 450, 475, 500, 525, and 550 K. The diffusion coefficients of the monolignol units and the water molecules were estimated from their mean-square displacement data using the Einstein relation and averaged over the two parallel trajectories.

### 3.4 Tensile Properties

Deformation simulations were performed using semi-isotropic pressure coupling, with two axes coupled, while the third one is in the direction of deformation. Parrinello–Rahman pressure coupling at 1 atm and velocity-rescaled temperature couplings were used. The studied deformation rates were 0.1, 1, and 10 m/s. The simulations were performed for 20 ns or longer for all systems, ensuring that the strain reached at least 20%. This ensured a sufficiently long interval to utilize a smoothing function for the pressure tensors, which tend to fluctuate.^36,37^ For each initial structure, deformation simulations were performed in all three orthogonal directions (*x*, *y*, *z*), and the average and standard deviation were calculated from these triplicates. Young’s Modulus (*E*) was calculated for each strain rate using Hooke’s law (eq 4), where σ is the stress and ε is the strain in the deformation direction. The strain interval 0.3–9.9% was used for calculating *E*.

$$E=\frac{\sigma }{\varepsilon }$$ 4

$$\nu \approx \frac{\Delta x_{P}}{\Delta x_{a}}$$ 5

Poisson’s ratio (ν) was determined using eq 5 where Δ*x*_a_ is the elongation of the simulation box in the deformation direction and Δ*x*_p_ is the corresponding contraction in the perpendicular direction. The Bulk Modulus (*K*) was subsequently calculated using Lame’s equation (eq 6).

$$K=\frac{-E}{6\left(\nu -\frac{1}{2}\right)}$$ 6

### 3.5 Energies

Energy analysis was used both to confirm the successful equilibration of the molecular systems and to assess how their stability was influenced by moisture and temperature. The latter simulations distinguished between bonded and nonbonded interactions. Bonded interactions, such as bond, angle, and dihedral energies, occur between chemically bound atoms. Nonbonded interactions, such as van der Waals forces (described using Lennard-Jones potentials) and electrostatic interactions (Coulomb forces), describe interactions between atoms that are not chemically bonded to each other.

## 4 Results

### 4.1 Experimental Section

Samples of the CTMP paper sheets pressed at different temperatures are presented in Figure 3, where “D” denotes sheets pressed in an almost dry state (dry, mc = 7 ± 1 wt %) and “W” represents moist-pressed sheets (wet, mc = 25 ± 1 wt %). Increasing the pressing temperature results in a darker paper sheet, indicating a certain degree of thermal degradation of the polymer components, which corresponds well to the degradation temperature intervals for the lignin (200 to 500 °C) and hemicellulose (220 to 315 °C).^42,43^ As shown in Figure 3, this degradation is generally more pronounced in the dry-pressed samples, where the sheet temperature during pressing is assumed to be higher. This is because, in the moist sheets, a significant portion of the applied heat is initially used to evaporate residual moisture before the temperature of the material can rise. In contrast, the dry sheets contain little to no moisture, allowing the sheet temperature to increase more rapidly during pressing.

**Figure 3:**
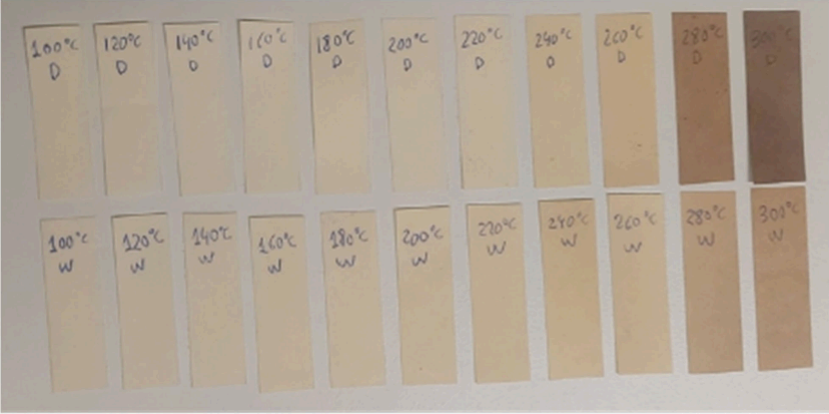
Examples of sheets pressed at varied temperatures ranging from 100 up to 300 °C. The sheets were initially either dry (D) or moist (W). Pressing was applied for 3 s at 3.5 MPa, followed by a posthold period of 6 s at 0.1 MPa.

SEM images of the cross sections of the paper sheets pressed at different temperatures (180 and 260 °C) and moisture contents are shown in Figure 4. The reference sheet (not hot-pressed) exhibits relatively high porosity with open surface pores and visible fiber lumens (Figure 4a). In the moist sample pressed at 180 °C (Figure 4b), the interfiber joints remain incomplete, with nano- or microscale gaps between adjacent fibers. These gaps arise from the limited moldability of moist fibers at this temperature level, which is due to the stiffness of the fiber walls. At 260 °C, a temperature which clearly exceeds the glass transition temperature of lignin,^20,44^ the moldability of the fiber wall increases (Figure 4c,d). This leads not only to the closure of interfiber pores and fiber lumens but also to the elimination of gaps in the joint regions. These changes are more pronounced when the fibers are moistened prior to hot-pressing (Figure 4d). In this case, the interfaces between contacting fibers become indistinguishable after hot-pressing, suggesting effective intermixing of the moist (and subsequently heated) surface polymers of adjacent fibers.

**Figure 4:**
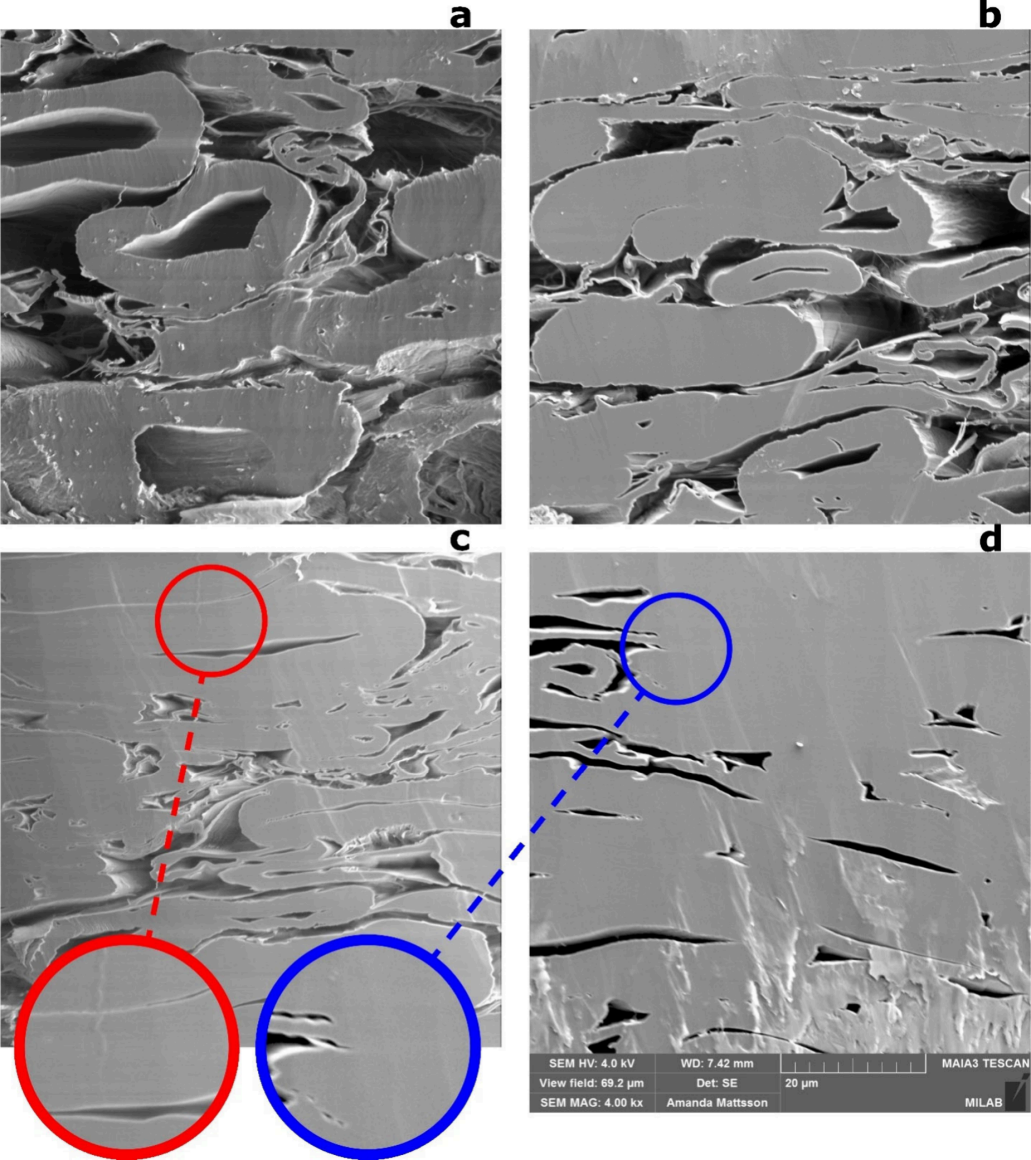
SEM images of paper sheet cross sections: (a) reference sample, (b) 180 °C moist, (c) 260 °C dry, and (d) 260 °C moist. Red and blue markings show areas where the interface (at 260 °C) between neighboring fibers becomes invisible under moist but not dry conditions.

The dry and wet tensile index (specific strength) of both moist-pressed (mc = 25 ± 1 wt %) and dry-pressed (mc = 7 ± 1 wt %) sheets are presented in Figure 5 for a range of pressing temperatures between 100 and 300 °C. The dry strength increased from 27.7 N m/g, without hot-pressing, to a maximum of 49.7 N m/g when pressing dry sheets at 280 °C (+79%) and to 62.9 N m/g when pressing moist sheets at 240 °C (+127%). Conversely, wet strength increased from zero, without hot pressing, to 21.2 N m/g and 25.7 N m/g when pressing at 300 °C dry and moist sheets, respectively. The pressing temperature has earlier been shown to be a dominant factor enhancing the mechanical properties of the sheets, with a particular emphasis on wet strength.^12^ Although both dry and wet strength increase with rising pressing temperature, this effect is particularly pronounced for wet strength above 180 °C. This threshold has earlier been attributed to lignin softening, which is expected to occur at a lower temperature in moist lignin compared to dry lignin.^44,45^ Furthermore, the moist-pressed sheets consistently exhibit higher dry and wet paper strength values than the dry-pressed ones, probably because the moisture in the sheets contributes to softening and to a changed distribution of hydrogen bonds within the matrix. On one hand, at low pressing temperatures (below 180 °C), no measurable wet strength was observed; dry strength was practically constant when pressing dry sheets, and a continuous increase is observed when pressing moist sheets. On the other hand, at very high pressing temperatures (above 260–280 °C), the dry strength tended to decrease, likely due to the degradation of hemicellulose and lignin,^42,43^ which makes the fibers more brittle. However, the exact temperature at which degradation begins to affect the strength depends on pressing time and sheet temperature.

**Figure 5:**
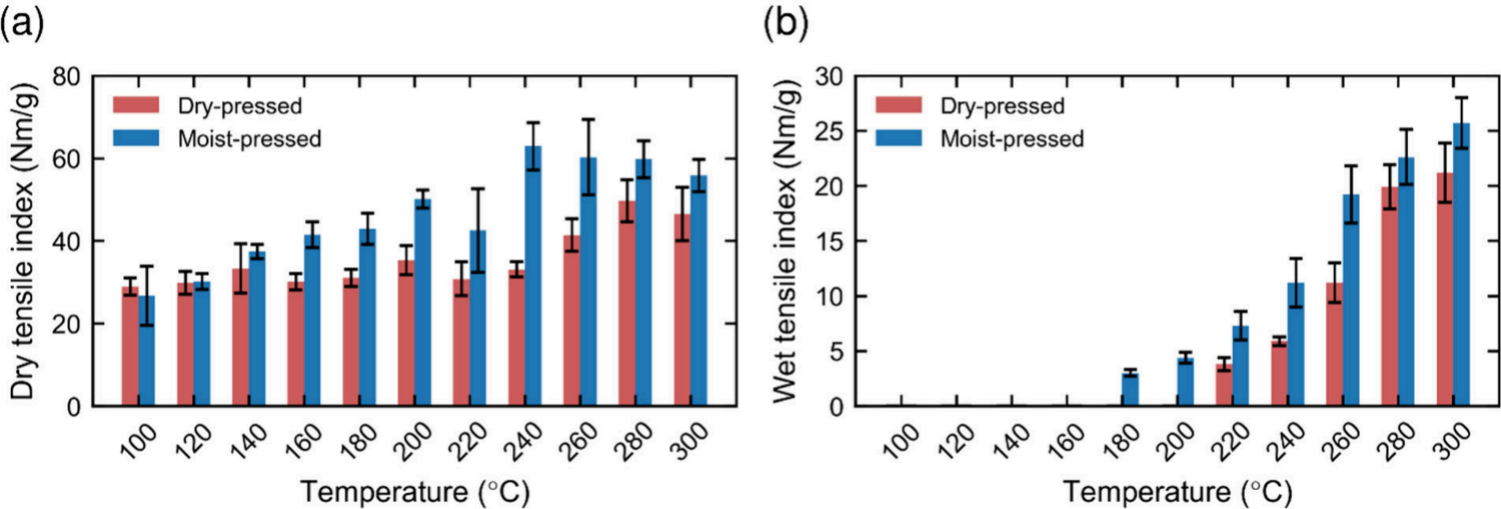
Dry (a) and wet (b) tensile indexes as a function of pressing temperature for dry and moist pressed sheets. The pressing was applied for 3 s at 3.5 MPa, followed by an after-hold of 6 s at 0.1 MPa. The dry tensile index for the unpressed reference is 27.7 N m/g, and the wet tensile index is zero (not measurable).

Using the wet tensile index data for varied pressing temperatures (100–300 °C), an activation energy was calculated, as shown in Figure 6. The energy is slightly higher for dry-pressed sheets (55 kJ/mol) than for moist-pressed sheets (42 kJ/mol), suggesting that moisture in the fibers facilitates lignin mobility. An even lower activation energy (26 kJ/mol) was previously reported for hot-pressing of various mechanical papers in pilot presses at a higher moisture content of 35–50 wt %.^46^ These values are compared in Section 4.2 with the corresponding activation energies for lignin diffusion from the MD simulations.

**Figure 6:**
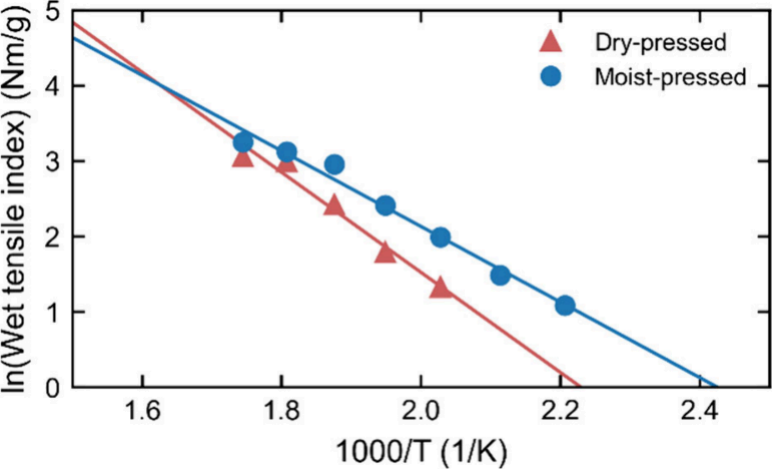
Logarithm of the wet tensile index plotted against the inverse temperature for both dry-pressed and moist-pressed sheets. The activation energy is obtained from the slope of the fitted lines.^46^

The effect of different moisture content levels on both dry and wet strength is shown in Figure 7 for two different pressing times, i.e., 1 and 5 s. Clearly, dry-pressed sheets do not achieve as high dry and wet strength as those moistened prior to hot-pressing. This seems to be related to the polymer intermixing at interfiber interfaces in the matrix, as shown in Figure 4. However, the strength values stay relatively constant, and the wet strength even decreases at the highest moisture content of 25 wt %. A moisture content that is too high in a sheet prior to hot-pressing can cause problems due to steam explosions. Additionally, the longer pressing time, i.e., 5 s, gives higher wet strength values, independently of the moisture content. This indicates that wet strength development is a time-dependent process and is faster for the moist samples than for the dry ones. Simultaneously, the dry strength seems to be unaffected by the pressing time. During hot-pressing, the densification of the sheets increases the number of interfiber joints.^12,47,48^ However, for dry fibers, the new joints are not necessarily as strong as the ones formed during sheet forming (Figure 4), which leads to less sensitivity of strength to the pressing time. Moreover, as seen in Figure 5a, the dry strength starts to decrease when the temperature exceeds 260 °C due to the degradation of lignin and hemicelluloses.

**Figure 7:**
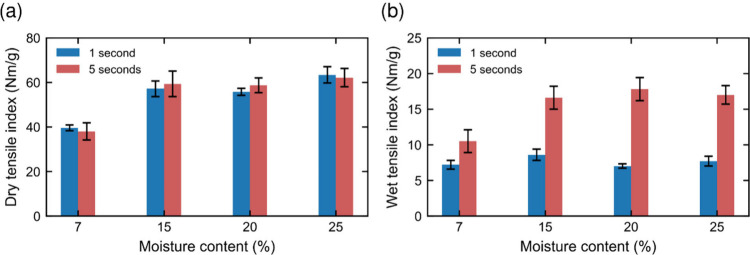
Tensile strength as a function of the moisture content in the sheets prior to pressing. Two different pressing times (1 and 5 s) were tested at a pressing temperature of 260 °C and a pressure of 3.5 MPa. (a) Dry tensile strength and (b) wet tensile strength.

### 4.2 Molecular Simulations

#### 4.2.1 Density and Glass Transition

Stepwise cooling simulations from a liquid state to a glassy state were used to study the effect of the temperature and moisture content on the lignin softening behavior. Figure 8 shows the results for the density and glass transition temperature.

**Figure 8:**
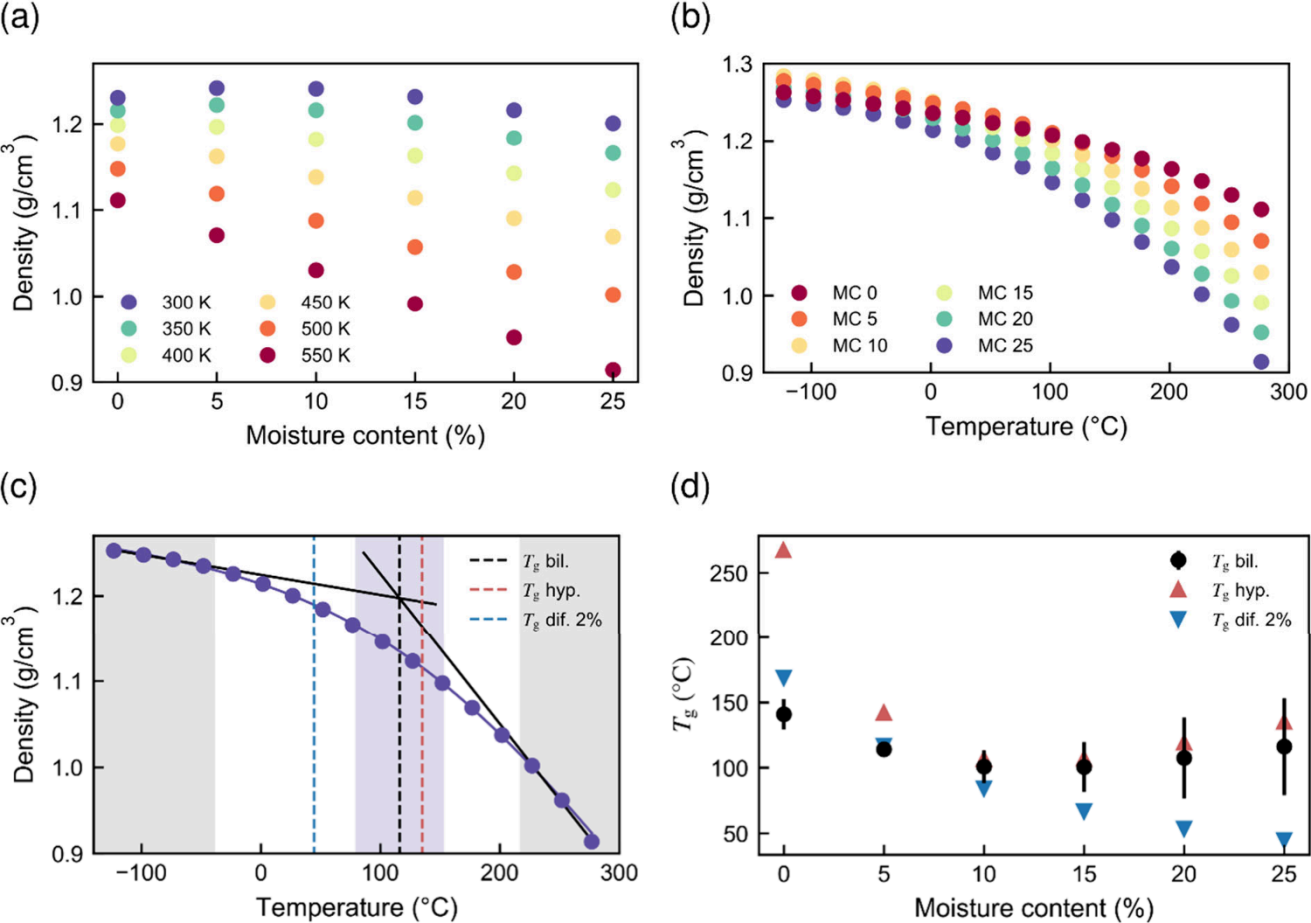
Results from the stepwise cooling simulations on neat lignin: (a) lignin density as a function of moisture content and temperature; (b) dilatometry curves obtained at different moisture contents (MC from 0 to 25 wt %); (c) example of *T*_g_ estimation based on bilinear, hyperbolic, and onset fitting methods (MC = 25 wt %); (d) *T*_g_ estimate as a function of moisture content obtained using the three methods.

Figure 8a shows the lignin density and its dependence on the moisture content and temperature. For the lowest temperature, 300 K (27 °C), we see an initial increase in density from 0 to 5 wt % moisture content, above which the density begins to decrease. Similar behavior has been observed in other biopolymer systems, such as amorphous cellulose. We attribute this to the rigidity of the lignin chains and the resulting subnanometer scale porosity, which means that a small amount of water can be accommodated without appreciable swelling.^49–51^ This initial density increase is also observable at 350 K (77 °C) but disappears at higher temperatures. This implies thermal softening of the systems. The density of the lignin model at room temperature, roughly 1.25 g/cm^3^, is at the lower end of the experimentally reported range of 1.2–1.5 g/cm^3^.^52^ The rather large variation is, at least partially, explained by the sensitivity of the lignin structure to the isolation method. The approximation of the uniform molecular weight used in the models is expected to cause packing inefficiency, which is reflected in the observed low density.

Figure 8b shows dilatometry curves, i.e., data for density versus temperature, obtained at different moisture contents. Well converged averaged density values were obtained by taking the average density from five simulations, where the densities of the individual simulations were computed as the mean over the simulation time. The averaged densities were subsequently used to calculate *T*_g_ as a function of moisture content, as described in Figure 8c.

Figure 8c illustrates how the bilinear, hyperbolic, and onset methods are used to estimate *T*_g_ for the model at 25 wt % moisture content. The low- and high-temperature linear fitting ranges, shown in gray, were selected to ensure that (i) they remained consistent across all models and (ii) the *T*_g_ estimate fell at least 100 °C above the lower fitting range and 100 °C below the upper fitting range. Hyperbolic fitting, on the other hand, was applied to all data points, with greater statistical weight assigned to the low-temperature end. The black dashed line represents the *T*_g_ estimate from bilinear fitting, with the blue highlighted area indicating the uncertainty of the estimate. The red and blue dashed lines correspond to estimates obtained using the hyperbolic *T*_g_ method and the *T*_g_-onset method (with a 2% threshold), respectively. The significantly lower *T*_g_ estimate obtained with the *T*_g_-onset method is indicative of a broad transition range. Lignin and many other biopolymers do not exhibit a sharp glass transition temperature (*T*_g_) but, rather, a broad temperature range, over which the transition gradually occurs. This makes the term *T*_g_ somewhat misleading for such polymers.

Figure 8d presents *T*_g_ estimates obtained at different moisture contents using all three methods. An initial decrease in *T*_g_ was observed for all methods up to a moisture content of 10–15 wt %. At higher moisture levels, the *T*_g_-onset value continued to decline, whereas the other *T*_g_ estimates unexpectedly increased due to the curvature at the high end of the dilatometry curves. The increasing thermal expansivity with moisture content, which causes nonlinear behavior at the high temperature end of the dilatometry curve, was identified as a major source of uncertainty, obscuring further moisture-related effects. This effect is presumably caused by pressure exerted by gaseous water trapped within the system.

Experimental measurements of dry spruce MWL lignin yielded *T*_g_ ≈ 145 °C with DMA (*E*′-onset) and *T*_g_ ≈ 155 °C with DSC,^53^ which closely matches our simulated *T*_g_ ≈ 145 °C at 0 wt % moisture. However, since the simulated *T*_g_ estimates are based on dilatometry at a cooling rate of 2.5 K/ns, they are not expected to be directly comparable to experimental values obtained at several orders of magnitude slower cooling rates. To approximate the effect of this cooling rate discrepancy, we calculated a theoretical shift factor using the Williams–Landel–Ferry (WLF) equation (eq 1) with parameters obtained from an additional series of simulations at varying cooling rates (see Supporting Information).^39,40^ This calculation predicted a temperature shift of approximately −41 °C when comparing the simulated cooling rate to a typical laboratory cooling rate of 10 °C/min. However, in the MD simulations, the higher cooling rate, which tends to increase *T*_g_, is counteracted by the shorter lignin chains, which lower *T*_g_ due to chain-end effects. As a result, reasonable *T*_g_ predictions were obtained, even without applying the WLF equation.

#### 4.2.2 Diffusion of Lignin and Water

Diffusivities of both lignin chains and water molecules were computed to investigate the influence of moisture transport through the paper and assess how the ductility of the lignin in the paper is influenced by the temperature and moisture content. Figure 9 shows the results, including diffusivities as a function of moisture content and reciprocal temperature, as well as diffusion activation energies. All properties were computed for both lignin repeat units and water molecules. The activation energies were obtained from the slopes of the Arrhenius plots using the temperature-dependent equation for the diffusivity: *D*(*T*) = *D*_0_*exp­(−*E*_d_/(R*T)).

**Figure 9:**
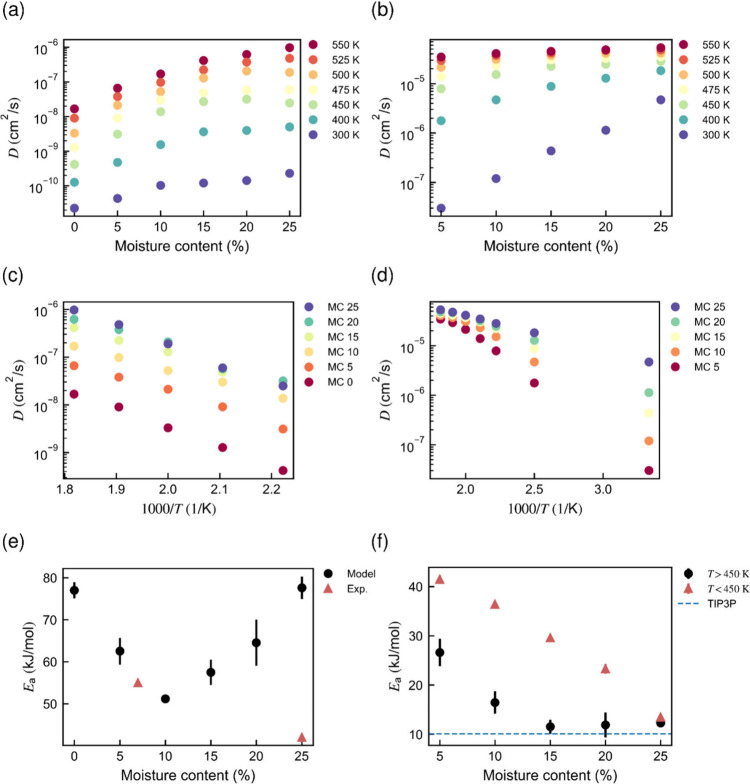
Results from isothermal diffusion simulations on neat lignin: (a) lignin and (b) water diffusivity versus moisture content for different temperatures; (c) lignin and (d) water diffusivity versus reciprocal temperature (Arrhenius plots); (e) lignin and (f) water diffusivity activation energies versus moisture content. In (e), the simulated value for lignin (Model) is compared with the experimental activation energy (Exp.) obtained from Figure 6. In (f), separate diffusion activation energies were computed above and below *T*_g_ due to the large curvature in the Arrhenius plot for water.

Lignin chain mobility (Figure 9a) increases exponentially with both moisture content and temperature, but the mobility increase with increasing temperature is more pronounced. For lignin diffusivity, a 25 °C temperature increase can approximately be substituted by a 5–10 wt % increase in moisture content. This can be compared to the wet strength measurements (Figure 5b), where a 20 °C increase (around 240 °C) produced a similar effect on wet tensile strength as the transition from dry-pressed to wet-pressed samples. However, in wood fibers, the water content is expected to be significantly lower in the lignin domains than in the (hemi)­cellulose regions,^28^ meaning that the change in moisture content within lignin may be smaller than in the bulk wood. The activation energy of lignin diffusion (Figure 9e) decreases up to 10 wt % moisture, after which it suddenly increases again. This could be a result of incipient phase separation between lignin and water at very high moisture contents, eventually beyond the solubility limit of water in lignin.^54^ At lower moisture levels, however, the activation energies for lignin diffusion closely resemble those observed for wet tensile strength, as shown in Figure 9e and further discussed in the conclusion section.

Water diffusivity in lignin (Figure 9b) also increases exponentially with both the temperature and moisture content. However, the moisture has a more pronounced effect on the water diffusivity than on the lignin diffusivity, particularly at room temperature. The activation energy of the water clearly decreases with increasing temperature (and moisture content), as observed for small molecule diffusion (Figure 9f). At lower water contents, water molecules tend to be more evenly dispersed within the lignin matrix, whereas at higher water contents, small regions of water clustering can form (Figure 10). These clusters are associated with higher water mobility compared to water molecules in close proximity to lignin, as the latter can form hydrogen bonds with lignin hydroxy groups or become confined due to steric hindrance.

**Figure 10:**
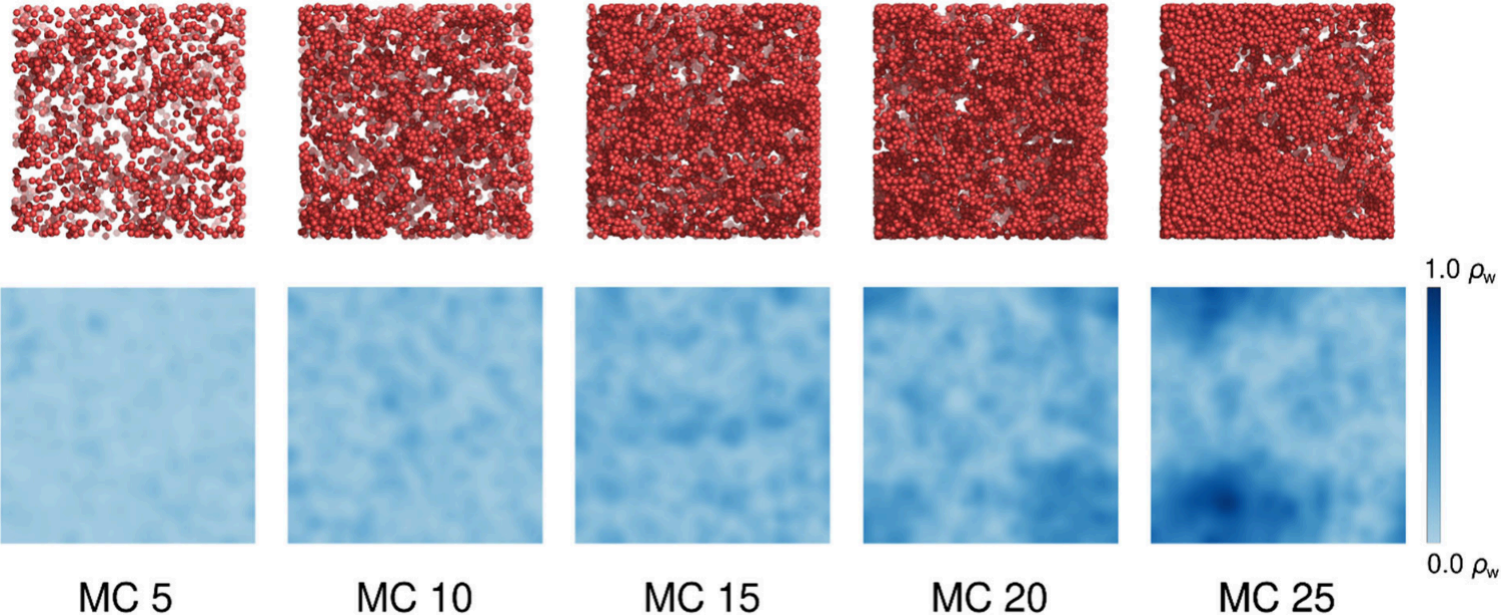
Visualization of the water distribution within the softwood lignin model at different moisture contents. Top row: Snapshots of water molecule locations shown as red spheres. Bottom row: Water mass density maps averaged in the through-thickness direction of the simulation domain and over 200 ns of simulation time.

This hypothesis was supported by analyzing the diffusivity distribution of individual water molecules, which revealed that, while most water molecules remain relatively stable, some exhibit significantly higher mobility (Figure 11). On very short time scales, a bimodal diffusivity distribution of individual water molecules is expected, with one group of slower water molecules located near the lignin matrix and another group of more mobile water molecules clustered in water pockets (Figure 11). On longer time scales, water molecules may transition between high- and low-mobility regions, thereby obscuring the bimodal distribution. Note that some diffusion simulations include temperatures above the boiling point of water. This is fortunately not a problem, since the used water model (TIP3P), which is optimized at 300 K, has a higher boiling point than 100 °C and therefore can be used at elevated temperatures.^36,37,55–57^

**Figure 11:**
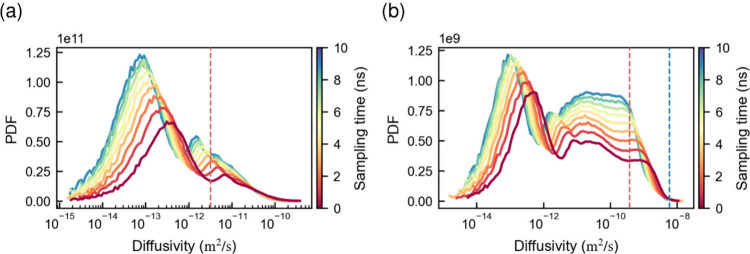
Distribution of molecular diffusivities of individual water molecules at (a) 5 and (b) 25 wt % moisture content. The effective diffusivities are calculated based on the distance traveled by each water molecule within different sampling times. The red dashed line indicates the diffusivity determined based on mean-squared displacement of the water molecules over 200 ns of simulated time. The blue dashed line indicates the self-diffusivity of liquid TIP3P water at 300 K.

Lignin is often considered to be hydrophobic due to its aromatic subunits. Therefore, the main mechanism limiting water mobility could be steric hindrance rather than electrostatic interactions. This is, however, not the entire story, as lignin also contains several hydroxy groups and ketones. Our simulations show that both lignin and water diffusivity display an exponential dependence on the moisture content (Figure 9a,b). This behavior resembles earlier findings for water diffusion within amorphous cellulose and hemicelluloses, as well as aggregated cellulose microfibril structures.^58,59^ It should also be noted that interactions between the lignin molecules and the larger hemicellulose molecules could, to some extent, impede the mobility of lignin.

#### 4.2.3 Energy

The stability of a molecular system can be assessed by analyzing its energy terms. For all lignin systems, the bonded energy terms (i.e., energies from bonds, angles, and dihedrals between chemically bound atoms) contributed positively to the total energy (Figure 12a), whereas the nonbonded terms (i.e., van der Waals energies and electrostatic interactions) contributed negatively (Figure 12b). This difference arises because nonbonded interactions stabilize the system by pulling atomic structures together, while bonded interactions become strained under compression, exerting a force that tends to expand the system. Both bonded and nonbonded energy terms increase strongly and nearly linearly with increasing temperature.

**Figure 12:**
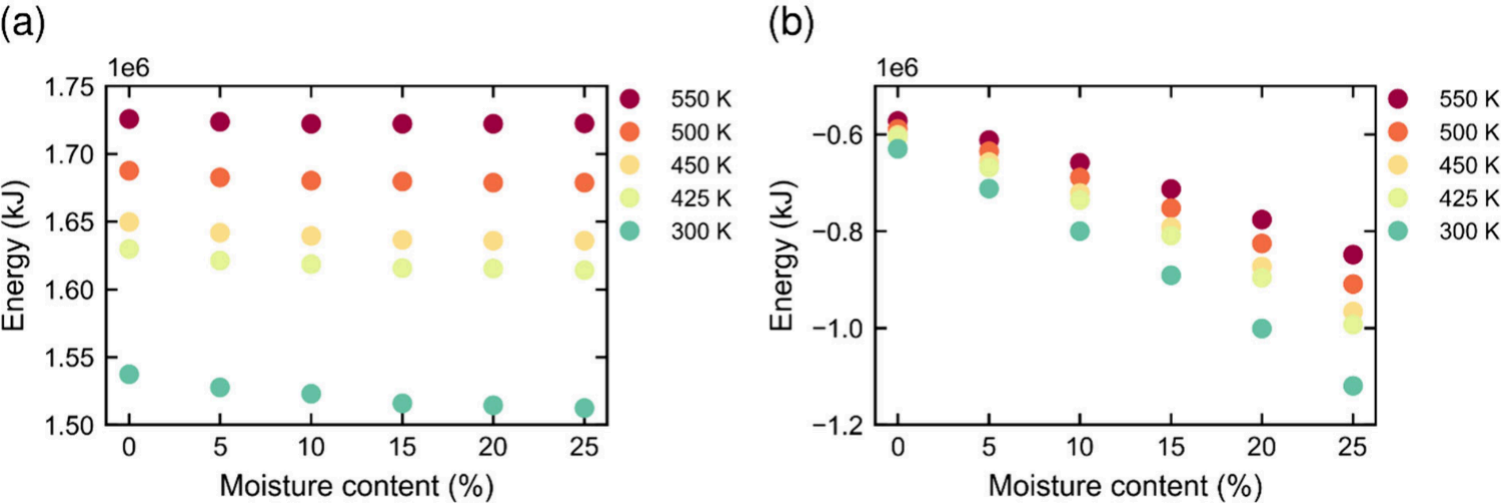
Total energies for all studied systems: (a) bonded terms and (b) nonbonded terms.

Moisture has a limited effect on the bonded energy terms. At low temperatures, the bonded energy decreases slightly with increasing moisture content but remains nearly constant at higher temperatures. This is because at elevated temperatures thermal energy is sufficient to overcome most barriers related to torsional rotations, reducing the influence of added water molecules.

The nonbonded energy decreases more significantly with increasing moisture content. This is due to stronger and more numerous electrostatic interactions, including hydrogen bonding and water–water and water–lignin interactions, leading to more stable structures. When the energy decreases because of a lower temperature, the material becomes more rigid. In contrast, when the decrease is due to added water, the material softens, as confirmed by our diffusion simulations. This is because, although water stabilizes the system by introducing more hydrogen bonds and other electrostatic interactions, these new interactions have a shorter average lifetime, resulting in a more dynamic molecular system. This explains the plasticizing effect of water and other plasticizers.

#### 4.2.4 Tensile Property Simulations

The mechanical properties of lignin are strongly dependent on the temperature and moisture content. With a simulation deformation rate of 0.1 m/s, Young’s modulus decreases rapidly with increasing temperature and moisture content (Figure 13a,b). The simulated Young’s modulus of lignin at room temperature and 10 wt % moisture content is *E* = 2.2 GPa, whereas experimental values for (Klason) lignin with 12 wt % moisture is reported by Cousins as *E* = 2.3 GPa.^60^ Although lignin extracted with the Klason method (i.e., treatment with concentrated sulfuric acid) differs somewhat from native softwood lignin, the close correlation between the *E* values is still notable. The bulk modulus typically also decreases but at a slower rate (Figure 13c), because it increases with rising Poisson’s ratio, which in turn is positively correlated with temperature and moisture content (Figure 13d). Poisson’s ratio fluctuates below 2% strain, and at higher strains, it decreases slightly as a function of strain due to orientation effects (Figure SI2).

**Figure 13:**
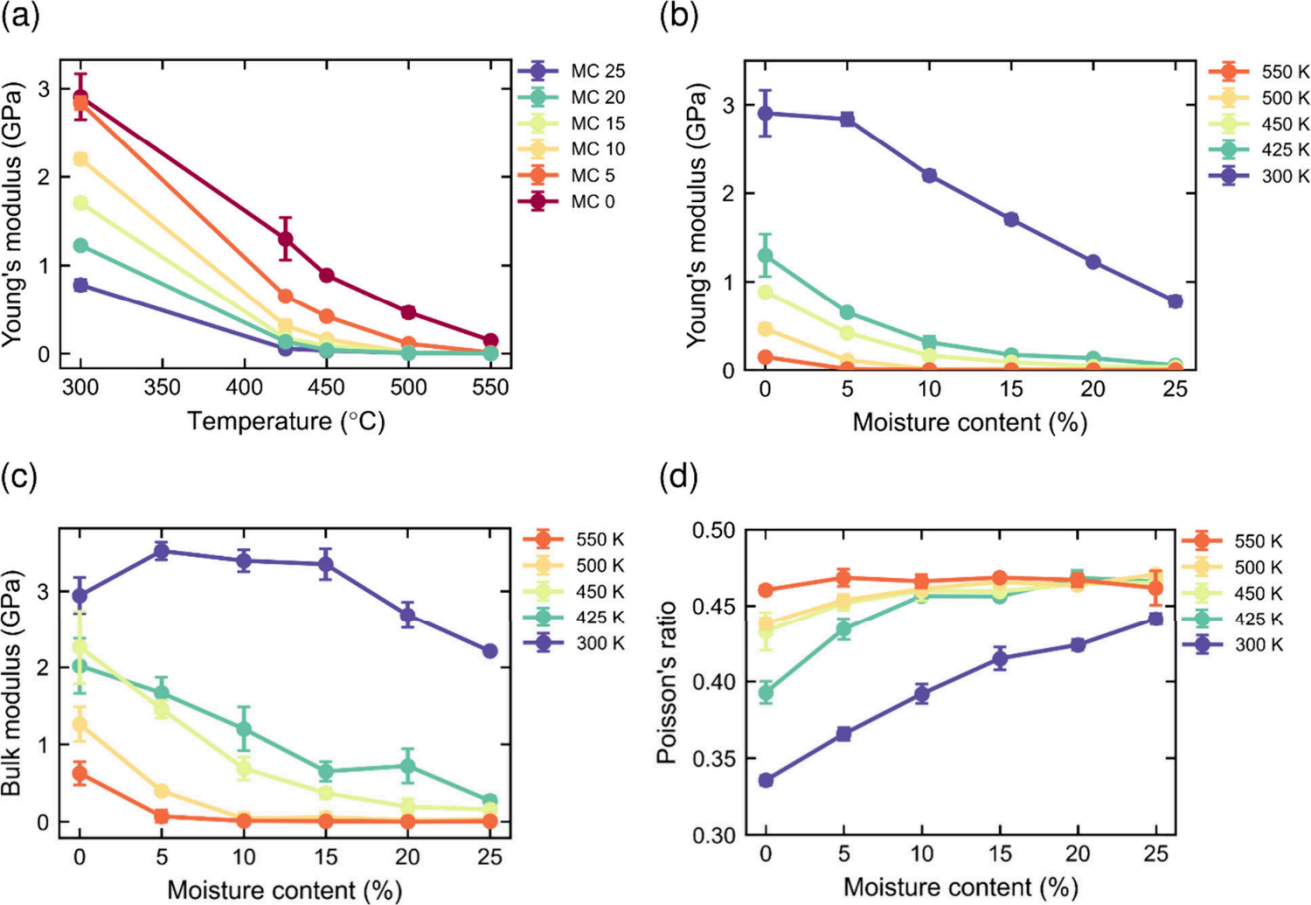
Mechanical properties for the systems using 0.1 m/s deformation rate: (a) Young’s modulus as a function of temperature, (b) Young’s modulus as a function of water content, (c) bulk modulus, and (d) Poisson’s ratio.

According to the simulations, lignin behaves as a rigid solid at room temperature. However, above approximately 150 °C, or even lower if the WLF-shift is considered, it gradually softens and becomes more deformable, especially at high moisture contents. At temperatures above 200 °C and moisture contents exceeding 10%, the simulated lignin transitions into a fluid-like state, making it highly deformable. Under these conditions, lignin can easily flow and rearrange, allowing it to solidify upon cooling and form a strong adhesive bond between cellulose fibrils on the surfaces of the bonded fibers. This behavior can explain our experimental results, where the wet tensile strength of the material increased drastically around 200 °C, particularly for samples with high moisture content (Figure 5). This change is further supported by microstructural observations with SEM, which show a distinct disappearance of gaps between fibers at high temperature and moisture content (Figure 4). Hemicellulose, which has a *T*_g_ in the same temperature range as lignin (220 °C at 0 wt % water and decreasing with increasing moisture content^20,61^ ) can however probably also co-contribute with the lignin to the “gluing” behavior.

The strain rates used in the simulations are much higher than those typically applied in laboratory settings. For a simulation box with a 21 nm side length, our deformation rate of 0.1 nm/ns corresponds to a strain rate of 5·10^6^ 1/s, which can be compared with the strain rate of 10^–4^ 1/s used in Cousins experiments. This may lead to an overestimation of the elastic modulus, as *E* increases with strain rate. At higher strain rates, atoms have less time to adjust their positions, which makes the material less ductile. However, the expected stiffening effect of high strain rates is often counterbalanced by other factors that soften the material, such as the use of smaller polymer molecules than those observed experimentally in polydisperse lignin materials. Extrapolation of mechanical data to experimental strain rates can eventually be used by applying the WLF equation (eq 1),^62^ but we prefer to report nonextrapolated data since some of the temperatures are outside of the valid range of the WLF equation. Nevertheless, it remains crucial to use sufficiently low strain rates to minimize the computational artifacts. For example, at the highest deformation rates (1 and 10 m/s), a small artificial peak in Young’s modulus is observed around 5 wt % moisture at 300 K, which is not present at the lower deformation rate (0.1 m/s) (Figure 13a). At very high strain rates, the water molecules do not have enough time to rearrange, fill cavities, and form stabilizing hydrogen bonds within the structure. This results in a stiffer, less plasticized system with premature void formation and a lower maximum strain at break.

## 5 Discussion

The comparison between the simulation results and experimental data showed several important trends that provide insight into the mechanisms by which lignin and water affect the thermoelastic properties of hot-pressed sheets.

One significant observation is the decrease in the simulated glass transition temperature of lignin with increasing moisture content, which is experimentally reflected as a marked increase in the wet tensile index with rising temperature. This aligns well with the known effect of water acting as a plasticizer, facilitating greater mobility for the lignin within the structure and thereby reducing the material stiffness. Interestingly, the activation energy determined from experimental wet strength values of dry-pressed sheets (mc = 7 wt %), 55 kJ/mol, was similar to the simulated activation energy for lignin diffusion at the same moisture content. However, this agreement was lost at higher moisture contents, likely due to the phase separation between lignin and water in the simulations.

It is also interesting to compare our simulated diffusivities for water and lignin with earlier simulation results obtained for cellulose microfibril bundles.^58^ According to Figure 8b, the diffusivity of water at room temperature (300 K) varies in the range of (2–500) × 10^–12^ m^2^/s depending on the moisture content (5–25 wt %). This is about 3 orders of magnitude higher than for lignin, (2–20) × 10^–15^ m^2^/s (Figure 8a), which is mainly attributed to the different molecular sizes. Both diffusivities are approximately 100 times larger at 500 K when fibers are almost dry (mc = 5 wt %). For cellulose microfibrils, the diffusion coefficient for water molecules is (2–100) × 10^–12^ m^2^/s in the same moisture range.^58^ Interestingly, this diffusion rate in the partly crystalline (hemi)­cellulose bundle structure is slightly lower than that in amorphous lignin. Thus, it can be concluded that lignin nanodomains in the fibers do not limit water diffusion more than the microfibril structure itself.

During hot pressing, fibers dry faster than lignin diffuses, so the lignin interdiffusion takes place in an environment with a gradually decreasing moisture content. At constant temperature, this means that the lignin interdiffusion rate slows over time. However, in practice, hot pressing causes the fiber temperature to increase when most of the moisture has evaporated, which compensates for this effect and allows the interdiffusion to continue effectively throughout the pressing period, provided the temperature is sufficiently high.

The experimental data for the wet tensile index was clearly lower than for the dry tensile index. This was reflected in the simulations, where the Young’s modulus decreased with increasing water content, particularly at room temperature. At elevated temperatures, the simulations showed a decrease to nearly negligible amounts at higher water contents, accompanied by increased mobility. This can be attributed to the reduced structural integrity of the lignin network, which we believe facilitates lignin migration along and across the fiber–fiber interfaces within the sheet, affecting the mechanical properties and making the material formable at somewhat lower temperatures. This is also evident in the SEM images, which reveal a denser structure at higher pressing temperatures and longer pressing times. We believe that, during this process, the lignin migrates and acts as a “glue” together with the hemicellulose, binding the pressed structures together, resulting in a more homogeneous and compact structure. This change in the physical structure subsequently affects the internal mobility of the material, where we expect that for the wet-pressed samples water is pressed out, except for the small amounts that get trapped within the structure.

The noticeable color shift in the sheets after hot pressing, indicating severe degradation, began to occur above 260 °C. This suggests a decrease in the molecular weight of the lignin, associated with the breakdown of the lignin and cellulosic structure within the sheets. In addition to degradation reactions, condensation reactions may also occur at elevated temperatures. These involve the elimination of water and the formation of new covalent bonds, contributing to an increased wet strength. However, such reactions can also lead to further darkening and increased brittleness of the material. This combination of degradation and condensation processes may explain some of the more significant changes in mechanical properties, such as a decrease in strength. The relationship between molecular degradation and mobility is important to consider, as it complicates direct comparisons between temperatures and explains why different results are obtained when hot pressing different types of paper sheets.

## 6 Conclusions

Based on molecular simulations and hot-pressing experiments, it is demonstrated that lignin in fibers plays a significant role in the thermoformability of paper sheets. Lignin has significant mobility at the elevated temperatures used for hot pressing. This mobility is increased with added moisture since water also acts like a plasticizer. After the sheet is cooled to room temperature, interdiffused lignin can (together with hemicellulose) glue neighboring cellulose microfibril and fiber interfaces together and thus significantly contribute to the mechanical properties of the sheet. This involves changes in both structural (e.g., polymer packing and density) and dynamical (e.g., diffusion and stress relaxation) properties of the material. The very distinct diffusion rates of water and lignin must be considered when optimizing material properties and energy use in a hot-pressing operation.

This is the first study that systematically correlates experimental strength of hot-pressed paper sheets with molecular simulations of lignin. However, our atomistic model for softwood lignin is not limited to modeling hot-pressing of paper and wood. It can also be used to assess other lignin applications, such as biobased binders and composites.

## Supplementary Material



## Notes

Images of the equipment used for hot-pressing, data for Poisson’s ratio, dilatometry data, and tables of added force field parameters and definitions (PDF)
