# Supplementary material for: Role of Lignin in Hot-Pressing of Paper: Insights from Molecular Simulations and Experiments
Source: Biomacromolecules. 2025 Aug 1;26(9):5965–78. doi: 10.1021/acs.biomac.5c00872 (PMC12421678; doi:10.1021/acs.biomac.5c00872)
Supplement: Supplementary file 1 [file bm5c00872_si_001.pdf]

Supplementary Information
Role of Lignin in Hot-Pressing of Paper: Insights from Molecular Simulations and Experiments

Patric Elf[1,4], Amanda Mattsson[2]*, Antti Paajanen[3], Jukka A. Ketoja[2,3], Gunilla Pettersson[2], Jose Luis Sanchez-Salvador[6], Angeles Blanco[6], Carlos Negro[6], Per Engstrand[2], Mikael S. Hedenqvist[1,4], Fritjof Nilsson[1,2,4]*

[1]School of Engineering Sciences in Chemistry, Biotechnology and Health, Fibre and Polymer Technology, KTH Royal Institute of Technology, SE-100 44 Stockholm, Sweden

[2]FSCN Research Centre, Mid Sweden University, 85170 Sundsvall, Sweden

[3]VTT Technical Research Centre of Finland Ltd, Box 1000, FI-02044 VTT, Espoo, Finland

[4]FibRe Centre for Lignocellulose-based Thermoplastics, KTH Royal Institute of Technology, SE-100 44 Stockholm, Sweden

[5]Department of Chemistry and Chemical Engineering, Chalmers University of Technology, SE-412 96 Gothenburg, Sweden

[6] Department of Chemical Engineering and Materials, Faculty of Chemistry. Universidad Complutense de Madrid, Avda. Complutense s/n, ES-280 40, Madrid, Spain

Figures

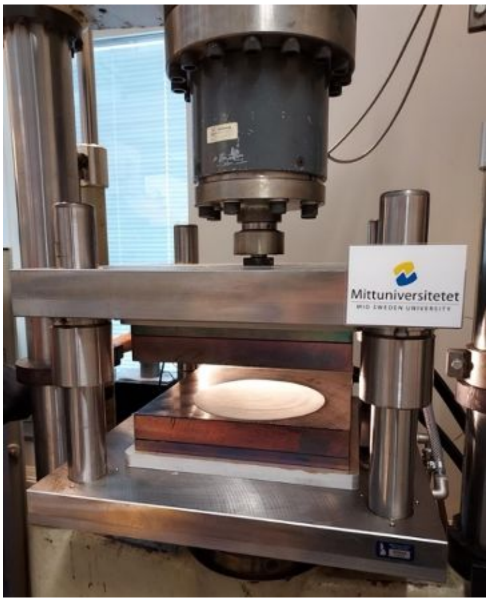

Figure SI1. Planar hot-pressing equipment at Mid Sweden University.

Figures for SI:

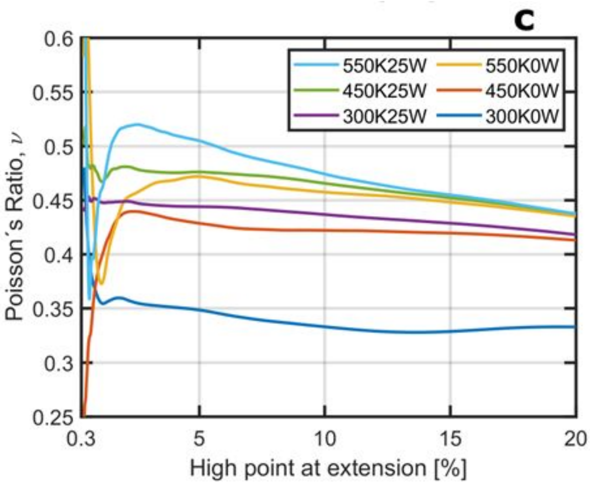

Figure SI2: Poissons ratio versus interval used for calculations.

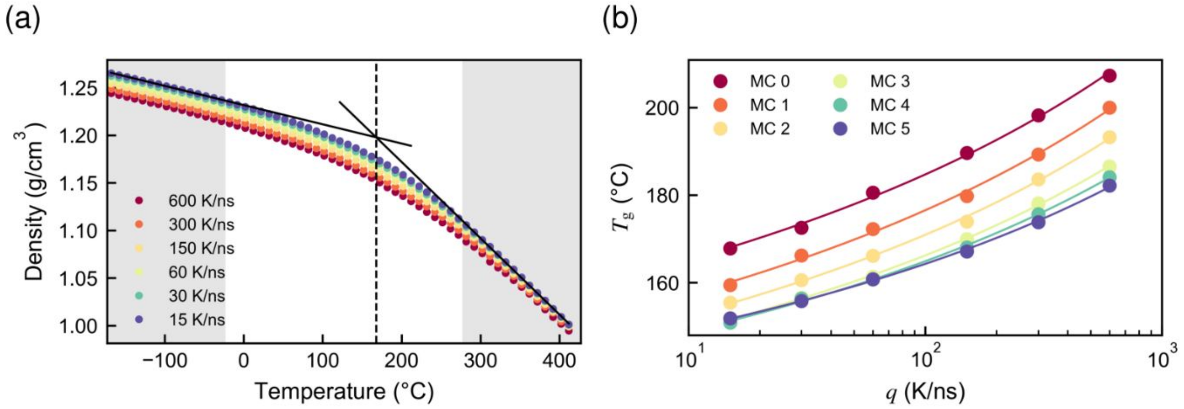

Figure S3: (a) Dilatometry curves obtained from the constant-rate cooling simulations at 0 wt.% moisture content; (b) WLF equation (solid lines) fitted against the T_g estimates obtained at different cooling rates and moisture contents (dots). In (a), the grey highlight indicates the asymptotic fitting regions, the solid black lines demonstrate a bilinear fit at the 15 K/ns cooling rate, and the dashed line shows the corresponding T_g estimate.

Tables

Table 1: Added bonded definitions, $V(\text{bond}) = K_r(r - r_0)^2$.

Atom types		K_r	r_0
CLG321	OLG301	360	1.415
CLG2R61	CLG321	230	1.49
CLG321	CLG321	222.5	1.53
CLG2O2	CLG321	200	1.522
CLG2O2	OLG301	700	1.23
CLG2O5	OLG2D4	700	1.23
CLG2O4	OLG311	700	1.21
CLG2O2	OLG2D4	700	1.21
CLG2O4	CLG311	250	1.5
CLG2O4	OLG2D4	700	1.21

Table 2: Added Angle definitions, $V(\text{angle}) = K\Theta(\Theta - \Theta_0)^2$, $V(\text{Urey-Bradley}) = K_{ub}(S\text{-}S_0)^2$.

Atom types			KΘ	$K\Theta_0$	K_{ub}	S_0
CLG311	OLG301	CLG321	95	109.7		
CLG2DC1	CLG321	OLG301	20	99		
OLG301	CLG321	HLGA2	45.9	108.89		
CLG311	CLG321	OLG301	75.7	110.1		
CLG2R61	CLG2R61	CLG321	45.8	120		
CLG2R61	CLG321	HLGA2	49.3	107.5		
CLG2R61	CLG321	CLG321	51.8	107.5		
CLG2O2	CLG321	CLG321	52	108		

CLG321	CLG2O2	OLG311	55	110.5		
CLG321	CLG2O2	OLG2D1	70	125	20	2.442
CLG2O2	CLG321	HLGA2	33	109.5	30	2.163
CLG321	CLG321	HLGA2	26.5	110.1	22.53	2.179
CLG2R61	CLG2O2	CLG321	20	116.5		
CLG2R61	CLG2O2	OLG301	70	121.3		
CLG321	CLG2O2	OLG301	75	122.2		
CLG321	CLG321	OLG311	75.7	110.1		
CLG321	CLG321	CLG321	58.35	113.6	11.16	2.561
CL2O2	CLG321	CLG2R61	51.8	107.5		
CLG321	CLG2O2	CLG321	35	115.6		
CLG321	CLG2O2	OLG2D4	75	122.2		
CLG2O2	CLG321	OLG311	112	111		
CLG2R61	CLG2O4	OLG311	75	126		
OLG311	CLG2O4	HLGR52	65	118		
CLG2O2	CLG321	CLG2R61	51.8	107.5		
CLG2R61	CLG2O2	OLG2D4	70	123.1	20	2.442
CLG2O4	CLG311	HLGA1	50	109.5		
CLG2O4	CLG311	OLG311	80	107		
CLG2R61	CLG2O4	CLG311	40	117.2		
CLG2R61	CLG2O4	OLG2D4	75	126		
CLG2O4	CLG311	CLG321	52	108		
CLG311	CLG2O4	OLG2D4	45	126		
CLG2O4	CLG311	CLG311	52	108		
CLG311	CLG2O4	OLG2D1	45	126		
CLG311	CLG2O4	HLGR52	65	116		
CLG2O4	CLG311	OLG301	70	109		

Table 3: Added dihedral definitions, V(dihedral) = $K_\chi(1 + \cos(n(\chi) - \delta))$.

Atom types				K_χ	n	δ
CLG2R61	CLG311	OLG301	CLG321	0.1	1	180
HLGA2	CLG321	OLG301	CLG311	0.284	3	0
CLG2DC1	CLG321	OLG301	CLG311	0.1	1	180
HLGA1	CLG311	OLG301	CLG321	0.284	3	0
CLG311	CLG311	OLG301	CLG321	0.4	1	0
CLG311	CLG311	OLG301	CLG321	0.49	3	0
CLG2DC1	CLG2DC1	CLG321	OLG301	0.7	3	0
HLGA4	CLG2DC1	CLG321	OLG301	0.2	3	0
OLG311	CLG311	CLG321	OLG301	0.2	3	0
CLG311	CLG321	OLG301	CLG311	0.24	1	0
CLG311	CLG321	OLG301	CLG311	0.29	2	0
CLG311	CLG321	OLG301	CLG311	0.02	3	0
CLG311	CLG311	CLG321	OLG301	0.2	3	180
HLGA1	CLG311	CLG321	OLG301	0.195	3	0
OLG301	CLG311	CLG321	OLG301	3.3808	2	180
CLG2R61	CLG2R61	CLG321	HLGA2	0.002	6	0

CLG2R61	CLG2R61	CLG321	CLG321	0.23	2	180
CLG321	CLG2R61	CLG2R61	HLGR61	2.4	2	180
CLG2R61	CLG2R61	CLG321	HLGA2	0.002	6	0
CLG2R61	CLG2R61	CLG321	CLG321	0.23	2	180
CLG2R6B	CLG2R61	CLG2R61	CLG321	3.1	2	180
CLG2R61	CLG321	CLG321	HLGA2	0.04	3	0
CLG2O2	CLG321	CLG321	CLG2R61	0.04	3	0
CLG321	CLG321	CLG2O2	OLG311	0.195	3	0
OLG2D1	CLG2O2	CLG321	CLG321	0.05	6	180
HLGA2	CLG321	CLG321	HLGA2	0.22	3	0
CLG2O2	CLG321	CLG321	HLGA2	0.195	3	0
CLG321	CLG2O2	OLG311	HLGP1	2.05	2	180
OLG311	CLG2O2	CLG321	HLGA2	0	6	180
OLG2D1	CLG2O2	CLG321	HLGA2	0	6	180
CLG2R61	CLG2R61	CLG2R61	CLG321	3.1	2	180
CLG321	CLG2O2	CLG2R61	CLG2R61	0.27	2	180
OLG301	CLG2O2	CLG2R61	CLG2R61	1.025	2	180
CLG2R61	CLG2O2	CLG321	HLGA2	0.1	3	0
CLG2R61	CLG2O5	CLG321	CLG321	0.4	1	0
CLG2O2	CLG321	CLG321	OLG311	0.2	3	0
OLG301	CLG2O2	CLG321	HLGA2	0	3	0
OLG302	CLG2O2	CLG321	CLG321	0.53	2	180
OLG301	CLG2O2	CLG321	CLG321	0.75	1	180
OLG301	CLG2O2	CLG321	CLG321	0.18	2	180
OLG301	CLG2O2	CLG321	CLG321	0.065	3	180
OLG301	CLG2O2	CLG321	CLG321	0.03	6	0
CLG321	CLG321	OLG311	HLGP1	1.13	1	0
CLG321	CLG321	OLG311	HLGP1	0.14	2	0
CLG321	CLG321	OLG311	HLGP1	0.24	3	0
OLG311	CLG321	CLG321	HLGA2	0.195	3	0
OLG301	CLG2O2	CLG321	CLG321	0.75	1	180
OLG301	CLG2O2	CLG321	CLG321	0.18	2	180
OLG301	CLG2O2	CLG321	CLG321	0.065	3	180
OLG301	CLG2O2	CLG321	CLG321	0.03	6	0
CLG2R61	CLG2O2	CLG321	CLG321	0.75	1	0
CLG2R61	CLG2O2	CLG321	CLG321	0.18	2	180
CLG2R61	CLG2O2	CLG321	CLG321	0.065	3	0
CLG2R61	CLG2O2	CLG321	CLG321	0.03	6	0
CLG2R61	CLG2R61	CLG321	CLG2O2	0.1	2	0
CLG321	CLG2O2	CG321	CLG2R61	0.3	1	0
CLG321	CLG2O2	CG321	CLG2R61	1	2	180
OLG2D4	CLG2O2	CG321	CLG2R61	0.85	1	0
CLG321	CLG2O2	CLG321	OLG311	0.75	1	0
CLG321	CLG2O2	CLG321	OLG311	0.18	2	180
CLG321	CLG2O2	CLG321	OLG311	0.065	3	0
CLG321	CLG2O2	CLG321	HLGA2	0.1	3	0

OLG2D4	CLG2O2	CLG321	HLGA2	0	3	0
CLG2O2	CLG321	OLG311	HLGP1	2.1	1	0
CLG2O2	CLG321	OLG311	HLGP1	1.4	2	0
CLG2O2	CLG321	OLG311	HLGP1	0.74	3	0
OLG2D4	CLG2O2	CLG321	OLG311	0	2	0
CLG2R61	CLG2R61	CLG2O4	OLG311	3.1	2	180
CLG2RC0	CLG2R61	CLG2R61	CLG2O4	3.1	2	180
CLG2RC0	CLG2R61	CLG2R61	CLG321	3.1	2	180
CLG2R61	CLG321	CLG2O2	CLG321	0.03819	2	0
CLG2R61	CLG321	CLG2O2	CLG321	0.03178	6	180
CLG2R61	CLG321	CLG2O2	OLG2D4	6.8277	2	180
CLG2R61	CLG2R61	CLG2O2	OLG2D4	6.8277	2	180
CLG2R67	CLG2R61	CLG2R61	CLG2O2	0.03819	2	0
CLG2R67	CLG2R61	CLG2R61	CLG2O2	0.03178	6	180
CLG321	CLG321	CLG2O2	OLG2D4	6.8277	2	180
CLG321	CLG321	CLG2O2	OLG2D4	6.8277	2	180
CLG2R61	CLG311	CLG311	CLG2O4	0.04	3	0
OLG2D1	CLG2O4	CLG311	CLG311	1.05	1	180
OLG2D1	CLG2O4	CLG311	CLG311	0.4	2	180
OLG2D1	CLG2O4	CLG311	CLG311	0.6	3	180
OLG2D1	CLG2O4	CLG311	CLG311	0.1	4	180
HLGR52	CLG2O4	CLG311	CLG311	0	3	180
CLG2O4	CLG311	CLG311	HLGA1	0.2	3	0
CLG2O4	CLG311	CLG311	OLG311	2	1	180
CLG2O4	CLG311	CLG311	OLG311	0.8	2	0
OLG2D1	CLG2O4	CLG311	HLGA1	0	3	180
HLGR52	CLG2O4	CLG311	HLGA1	0	3	180
OLG311	CLG311	CLG2O4	OLG2D1	0.2	3	0
OLG311	CLG311	CLG2O4	HLGR52	0.195	3	0
CLG2O3	CLG311	OLG311	HLGP1	0.3	1	0
CLG2O3	CLG311	OLG311	HLGP1	0.3	2	180
CLG2O3	CLG311	OLG311	HLGP1	0.4	3	0
CLG2O4	CLG311	OLG301	CLG2R6B	0.91	1	180
CLG2O4	CLG311	OLG301	CLG2R61	0.5	2	0
OLG2D4	CLG2O4	CLG311	OLG301	0.1	1	0
OLG2D4	CLG2O4	CLG311	OLG301	0.98	2	180
CLG2R61	CLG2O4	CLG311	OLG301	0	2	180
CLG311	CLG2O4	CLG2R61	CLG2R61	1.585	2	180
OLG2D4	CLG2O4	CLG2R61	CLG2R61	1.585	2	180
CLG2O4	CLG2R61	CLG2R61	CLG2R67	3.1	2	180
CLG2R61	CLG2O4	CLG311	CLG321	0.4	1	0
CLG2R61	CLG2O4	CLG311	CLG321	0.17	2	180
CLG2R61	CLG2O4	CLG311	CLG321	0.13	3	180
CLG2R61	CLG2O4	CLG311	HLGA1	0	1	180
CLG2O4	CLG311	CLG321	OLG311	0.2	3	0
CLG2O4	CLG311	CLG321	HLGA2	0.2	3	0

OLG2D4	CLG2O4	CLG311	CLG321	0.75	1	180
OLG2D4	CLG2O4	CLG311	CLG321	0.18	2	180
OLG2D4	CLG2O4	CLG311	CLG321	0.065	3	180
OLG2D4	CLG2O4	CLG311	CLG321	0.03	6	0
OLG2D4	CLG2O4	CLG311	HLGA1	0	1	0
CLG2O4	CLG311	OLG311	HLGP1	0.35	1	0
CLG2O4	CLG311	OLG311	HLGP1	0.37	2	0
CLG2O4	CLG311	OLG311	HLGP1	0.01	3	180
